# Role of oxidative stress in female reproduction

**DOI:** 10.1186/1477-7827-3-28

**Published:** 2005-07-14

**Authors:** Ashok Agarwal, Sajal Gupta, Rakesh K Sharma

**Affiliations:** 1 Center for Advanced Research in Human Reproduction, Infertility, and Sexual Function, Glickman Urological Institute and Department of Obstetrics-Gynecology; The Cleveland Clinic Foundation, Cleveland, Ohio 44195, USA

## Abstract

In a healthy body, ROS (reactive oxygen species) and antioxidants remain in balance. When the balance is disrupted towards an overabundance of ROS, oxidative stress (OS) occurs. OS influences the entire reproductive lifespan of a woman and even thereafter (i.e. menopause). OS results from an imbalance between prooxidants (free radical species) and the body's scavenging ability (antioxidants). ROS are a double-edged sword – they serve as key signal molecules in physiological processes but also have a role in pathological processes involving the female reproductive tract. ROS affect multiple physiological processes from oocyte maturation to fertilization, embryo development and pregnancy. It has been suggested that OS modulates the age-related decline in fertility. It plays a role during pregnancy and normal parturition and in initiation of preterm labor. Most ovarian cancers appear in the surface epithelium, and repetitive ovulation has been thought to be a causative factor. Ovulation-induced oxidative base damage and damage to DNA of the ovarian epithelium can be prevented by antioxidants. There is growing literature on the effects of OS in female reproduction with involvement in the pathophsiology of preeclampsia, hydatidiform mole, free radical-induced birth defects and other situations such as abortions. Numerous studies have shown that OS plays a role in the pathoysiology of infertility and assisted fertility. There is some evidence of its role in endometriosis, tubal and peritoneal factor infertility and unexplained infertility. This article reviews the role OS plays in normal cycling ovaries, follicular development and cyclical endometrial changes. It also discusses OS-related female infertility and how it influences the outcomes of assisted reproductive techniques. The review comprehensively explores the literature for evidence of the role of oxidative stress in conditions such as abortions, preeclampsia, hydatidiform mole, fetal embryopathies, preterm labour and preeclampsia and gestational diabetes. The review also addresses the growing literature on the role of nitric oxide species in female reproduction. The involvement of nitric oxide species in regulation of endometrial and ovarian function, etiopathogenesis of endometriosis, and maintenance of uterine quiescence, initiation of labour and ripening of cervix at parturition is discussed. Complex interplay between cytokines and oxidative stress in the etiology of female reproductive disorders is discussed. Oxidant status of the cell modulates angiogenesis, which is critical for follicular growth, corpus luteum formation endometrial differentiation and embryonic growth is also highlighted in the review. Strategies to overcome oxidative stress and enhance fertility, both natural and assisted are delineated. Early interventions being investigated for prevention of preeclampsia are enumerated. Trials investigating combination intervention strategy of vitamin E and vitamin C supplementation in preventing preeclampsia are highlighted. Antioxidants are powerful and there are few trials investigating antioxidant supplementation in female reproduction. However, before clinicians recommend antioxidants, randomized controlled trials with sufficient power are necessary to prove the efficacy of antioxidant supplementation in disorders of female reproduction. Serial measurement of oxidative stress biomarkers in longitudinal studies may help delineate the etiology of some of the diosorders in female reproduction such as preeclampsia.

## Review

### 1. Oxidative Stress

#### 1.1 Free radicals

**Figure 1:**
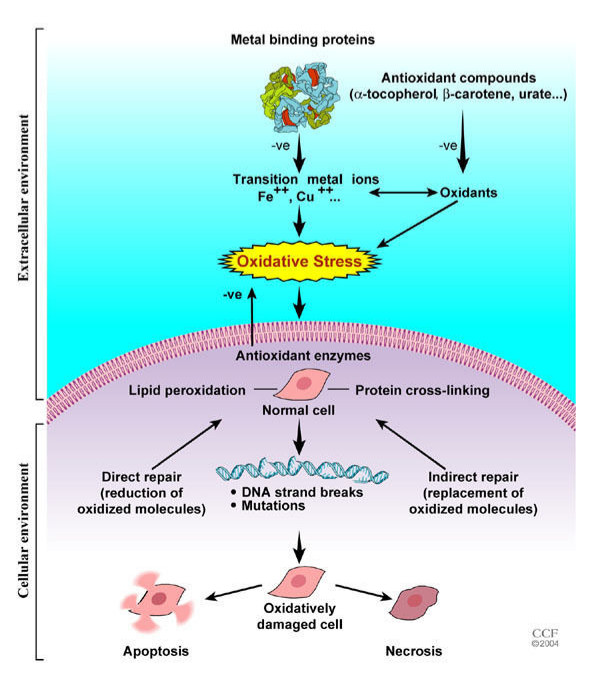
Mechanisms of oxidative stress-induced cell damage.

Free radical species are unstable and highly reactive. They become stable by acquiring electrons from nucleic acids, lipids, proteins, carbohydrates or any nearby molecule causing a cascade of chain reactions resulting in cellular damage and disease [1-4], figure 1) . There are two major types of free radical species: reactive oxygen species (ROS) and reactive nitrogen species (NOS).

#### 1.2 Reactive oxygen species

The three major types of ROS are: superoxide (O_2_^•-^), hydrogen peroxide (H_2_O_2_), hydroxyl (OH^•^). The superoxide radical is formed when electrons leak from the electron transport chain [5]. The dismutation of superoxide results in the formation of hydrogen peroxide. The hydroxyl ion is highly reactive and can modify purines and pyrimidines and cause strand breaks resulting in DNA damage [6]. Some oxidase enzymes can directly generate the hydrogen peroxide radical.

ROS have been implicated in more than 100 diseases [7-10]. They have a physiological and pathological role in the female reproductive tract. Numerous animal and human studies have demonstrated the presence of ROS in the female reproductive tract: ovaries, [11-15], fallopian tubes [16] and embryos [17]. ROS is involved in the modulation of an entire spectrum of physiological reproductive functions such as oocyte maturation, ovarian steroidogenesis, corpus luteal function and luteolysis [11,12,18]. ROS-related female fertility disorders may have common etiopathogenic mechanisms. ROS may also originate from embryo metabolism and from its surroundings.

#### 1.3 Reactive nitrogen species

Nitric oxide (NO) is synthesized during the enzymatic conversion of L-arginine to L-citrulline by nitric oxide synthase (NOS) [19-21]. With an unpaired electron, NO, which is a highly reactive free radical, damages proteins, carbohydrates, nucleotides and lipids and, together with other inflammatory mediators, results in cell and tissue damage, low-grade, sterile inflammation and adhesions [20]. NO potently relaxes arterial and venous smooth muscles and, less strongly, inhibits platelet aggregation and adhesion. NO donors, acting as vasodilating agents, are therefore a possible therapeutic approach [22]. NO acts in a variety of tissues to regulate a diverse range of physiological processes, but excess of NO can be toxic [1,20,21,23].

Reactive nitrogen species have been associated with asthma, ischemic/reperfusion injury, septic shock and atherosclerosis [24-27]. The two common examples of reactive nitrogen species are nitric oxide (NO) and nitrogen dioxide [1,3]. NO is produced by the enzyme NO synthase. There are 3 types of nitric oxide synthase (NOS) isoenzymes in mammals involving endothelial NO synthase (NO synthase 3), neuronal NO synthase (NO synthase 1) and inducible NO synthase (NO synthase 2). Neuronal NO synthase (nNOS) and endothelial NO synthase (eNOS) are constitutive NO synthases, and responsible for the continuous basal release of NO. Inducible NO synthase (iNOS) is present in mononuclear phagocytes (monocytes and macrophages) and produces a large amount of NO. This is expressed in response to proinflammatory cytokines and lipopolysaccharides [21,23,28]. Inducible NO synthase is activated by cytokines such as, interleukin-1, and TNF-α and lipopolysaccharides. Endothelial NO synthase is expressed in thecal cells, granulosa cells, and the surface of oocyte during the follicular development. In pathological conditions, inducible NO synthase might play a major role in NO production. In most organs, inducible NO synthase is expressed only in response to immunological stimuli [29].

#### 1.4 Antioxidants

Under normal conditions, scavenging molecules known as antioxidants convert ROS to H_2_O to prevent overproduction of ROS. There are two types of antioxidants in the human body: enzymatic antioxidants and non-enzymatic antioxidants [1,3].

#### 1.5 Enzymatic antioxidants

Enzymatic antioxidants are also known as natural antioxidants, they neutralize excessive ROS and prevent it from damaging the cellular structure. Enzymatic antioxidants are composed of superoxide dismutase, catalase, glutathione peroxidase and glutathione reductase, which also causes reduction of hydrogen peroxide to water and alcohol.

#### 1.6 Non-enzymatic antioxidants

Non-enzymatic antioxidants are also known as synthetic antioxidants or dietary supplements. The body's complex antioxidant system is influenced by dietary intake of antioxidant vitamins and minerals such as vitamin C, vitamin E, selenium, zinc, taurine, hypotaurine, glutathione, beta carotene, and carotene [1-3,30]. Vitamin C is a chain breaking antioxidant that stops the propagation of the peroxidative process. Vitamin C also helps recycle oxidized vitamin E and glutathione [31]. Taurine, hypotaurine and transferrin are mainly found in the tubal and follicular fluid where they protect the embryo from OS [17]. Glutathione is present in the oocyte and tubal fluid and has a role in improving the development of the zygote beyond the 2-cell block to the morula or the blastocyst stage [32].

#### 1.7. Oxidative stress in female reproduction

Cells have developed a wide range of antioxidants systems to limit production of ROS, inactivate them and repair cell damage [1-3,33]. OS influences the entire reproductive span of women's life and even thereafter (i.e. menopause). It has been suggested that the age-related decline in fertility is modulated by OS [34]. It plays a role during pregnancy [35] and normal parturition [36,37] and in initiation of preterm labor [38,39]. The pathological effects are exerted by various mechanisms including lipid damage, inhibition of protein synthesis, and depletion of ATP [40]. There is some understanding of how ROS affect a variety of physiologic functions (i.e. oocyte maturation, ovarian steroidogenesis, ovulation, implantation, formation of blastocyst, luteolysis and luteal maintenance in pregnancy) [14,15,18,19,41].

ROS are a double-edged sword – they serve as key signal molecules in physiological processes but also have a role in pathological processes involving the female reproductive tract. Since the balance is maintained by the presence of adequate amounts of antioxidants, measuring levels of the antioxidants, individually or as total antioxidant capacity (TAC), has also been examined [15,18,42,43]. Superoxide dismutase (SOD) enzymes, Copper-Zinc SOD (Cu-Zn SOD) and Manganese superoxide dismutase (MnSoD) have been localized in the granulose and thecal cells of the growing follicle. Selenium dependent glutathione peroxidase activity has been demonstrated in the follicular fluid and serum of patients undergoing IVF. The expression profiles of the transcripts of the antioxidant enzymes such as superoxide dismutase, glutathione peroxidase and gamma-glutamylcysteine synthetase in both human and mouse oviducts and oocytes have also been examined [16]. There is growing literature on the effects of OS in the female reproduction with involvement in the pathophsiology of pre-eclampsia [44,45], hydatidiform mole [46-48], free radical-induced birth defects [49] and other situations such as abortions [50].

#### 1.8 Measurement of oxidative stress

The presence of ROS and antioxidants in the female reproductive tract has been demonstrated by various methodologies in animal and human studies. A number of OS biomarkers have been investigated including superoxide dismutase, glutathione peroxidase, conjugated dienes, lipid peroxides, thiobarbituric acid reactive substances, glutaredoxin, oxidative DNA adducts, follicular fluid, NO and TAC [12,15,16,19,29,42,51-58] (Table 1).

**Table 1:** Oxidative stress biomarkers in female reproductive tract

**Study**	**Biomarker**	**Methodology**	**Measurement units**
Sugino et al [41]	Enzymatic antioxidants: Cu SOD, Mn SOD, catalase, glutathione peroxidase	Reverse transcription-polymerase chain reaction	cDNA sequences
Attaran et al [4]	Total antioxidant capacity	Enhanced chemiluminescence assay	Trolox equivalents
Jozwik et al [15]	Lipid peroxides; Malondialdehyde, conjugated dienes, Thiobarbituric acid reactive substances.	Thiobarbituric acid method	Micromole of malondialdehyde/L
Seino et al [52].	Oxidative DNA adducts	8-hydroxy 2-deoxyguanosine	Immunocytochemical staining

Metabolites of NO (nitrite and nitrate) in peritoneal fluid are determined by nitrate reductase and the Griess reaction [20,23]. Total NO (nitrite and nitrate) levels in the serum and follicular fluid assay of NO are measured via a rapid-response chemiluminescence analyzer [29]. Various biomarkers of oxidative stress have been determined in the placenta by immunohistochemistry or western blot analysis (Table 2). Oxidative DNA adducts 8-hydroxy 2-deoxyguanosine-have been studied by immunostaining in placenta [45], in patients with IUGR (intrauterine growth retardation) and patients with preeclampsia and IUGR [45]. The basal levels of ROS in the leukocytes in whole blood can be determined using the dihydroethidium and dichlorodihydrofluorescein-diacetate probes (Table 2).

**Table 2:** Measurement of biomarkers of oxidative stress in pregnancy

**Study**	**Biomarkers**	**Methodology**	**Measurement units**
Jauniaux et al [59]	Immunohistochemistry	Heat shock protein 70, hydroxynenal, nitrotyrosine residues.	Fluorescence intensity
Wang et al, Djordjevic et al [60, 61]	Antioxidant enzyme activity assays	Total SOD activity, catalase activity, glutathione peroxidase activity, reduced glutathione assay	Change in optical density/minutes/mg protein
Wiktor et al [62]	Oxidative DNA adducts	8-hydroxy-2 deoxyguanosine	Micromoles/mole of 2-deoxy guanosine
Holthe et al [63]	Superoxide anion, hydrogen peroxide and peroxynitrite	Dihydrethidium probe Dichlorodihydrofluorescein Dihydrorhodamine 123 Spectrophotometry/flow cytometry	Nanomoles/10 min/10^6 ^cells
Ishihara et al [64]	Lipid peroxidation products	Isoprostane, Urinary 8-epi-prostaglandin F2lpha, assayed by gas chromatography/mass spectrophotometer analysis	pg/mg of creatinine
Vaisanen-Tommiska et al [65]	Nitric oxide; Greiss reaction	Stable end products: nitrite/nitrate	Fluorometric assay, results expressed as NOx (sum of converted nitrite and very small amount of nitrite in serum).
Buhimishi et al [66]	Plasma and red blood cell glutathione content	Colorimetric assay	Nanomoles/mgm of haemoglobin

### 2. Oxidative Stress & Female Infertility

Infertility is a disease defined as "the inability to conceive following 12 or more months of unprotected sex before an investigation is undertaken unless the medical history and physical findings dictate earlier evaluation and and treatment [67]. The prevalence of female infertility ranges from 7% to 28%, depending on the age of the woman. In general, an estimated 84% of couples conceive after 1 year of intercourse, and 92% of the couples conceive after 2 years [68]. Although the frequency and origin of different forms of infertility varies, 40%–50% of the etiology of infertility studied is due to female causes [69].

A primary diagnosis of male factor infertility is made in 30% of infertile couples. Combined female and male factor infertility is responsible for 20%–30% of cases. Finally, unexplained infertility affects 15% of couples [70]. If the results of a standard infertility examination are normal, a diagnosis of unexplained or idiopathic infertility is assigned [70]. Data from the National Survey for Family Growth indicate that the number of women with impaired fecundity has increased from 1982 to 1995, an increase of 35% in the number of women. Approximately 1.3 million American couples receive medical advice or treatment for infertility every year [71]. OS has a role in etiopathogenesis of endometriosis, tubal factor infertility, and unexplained infertility. Impact of OS on ART is discussed in further sections.

#### 2.1 Pathophysiology of oxidative stress in female reproduction

Oxygen toxicity is an inherent challenge to aerobic life [72]. ROS can modulate cellular functions, and OS can impair the intracellular milieu resulting in diseased cells or endangered cell survival. The role of ROS in various diseases of the female reproductive tract has been investigated. ROS can affect a variety of physiological functions in the reproductive tract, and excessive levels can result in precipitous pathologies affecting female reproduction. The oxidant status can influence early embryo development by modifying the key transcription factors and hence modifying gene expression [73]. Concentrations of ROS may also play a major role both in the implantation and fertilization of eggs [72]. There is an increased interest to examine the role of OS in female reproduction because it may be a major link in the infertility puzzle as well as in some reproductive organ diseases such as endometriosis. Recently, OS has been reported to have an important role in the normal functioning of the female reproductive system and in the pathogenesis of female infertility [33,74].

#### 2.2 Cytokines, oxidative stress and female reproduction

The control of ovarian stromal cells and germ cell function is a diverse paradigm and oxidative stress may be one of the modulators of ovarian germ cell and stromal cell physiology. A number of autocrine and paracrine factors affect the modulation of various ovarian functions and steroidogenesis. Cytokines are polypeptides or glycoproteins secreted into the extra cellular compartment by the leukocytes [75]. Mammalian ovulation or follicular rupture was proposed to result from the vascular changes and the proteolytic cascade [54]. The cross talk between these two cascades is mediated by cytokines, vascular endothelial growth factor (VEGF), and ROS (both reactive nitrogen and oxygen radicals). Interleukin-1β causes nitrite to accumulate in rat ovarian dispersates, demonstrating the close interaction between cytokines and NOS [76]. OS and cytokines are proposed to be interlinked and act as intercellular and intracellular messengers in the ovary. A number of investigators have investigated the synthesis of NOS and ROS in the ovaries [21,55,58].

Defective placentation leads to placental hypoxia and reperfusion injury due to ischemia and the resultant OS triggers the release of cytokines and prostaglandins, which results in endothelial cell dysfunction and plays an important role in the development of pre-eclampsia [77,78]. TNF-α a plasma cytokine, has been demonstrated to cause the endothelial cell injury [79]. A link between OS and expression of cytokine receptors in the cytotrophoblast, vascular smooth muscle cells and endometrial cells has also been proposed, further establishing that hyperactivation of ROS may result in pre-eclampsia [80].

The activation of mononuclear phagocytes can be triggered in endometriosis by a number of factors including damaged red blood cells and the apoptotic endometrial cells. A positive correlation between concentrations of tumor necrosis factor (TNF)-α in the peritoneal fluid and endometriosis has been reported [75]. Cytokines released by the macrophages influence the redox status of the ectopic endometrium in patients with endometriosis [81]. Superoxide dismutase, glutathione peroxidase activity and lipid peroxidation levels were measured in ectopic endometrial tissue obtained from ovarian endometriomas. Superoxide dismutase activity was found to be significantly higher in the ectopic endometrium than in eutopic endometrium, and a positive correlation was seen between malondialdehyde levels and plasma 17-beta estradiol levels. TNF-α has been shown to cause up regulation of expression of Manganese (Mn) superoxide dismutase in the endometrium in vitro [82]. The antioxidant MnSOD neutralizes superoxide anions generated by cytokine TNF-α. This is a self protective mechanism against TNF-α induced oxidative stress. Estrogen and progesterone withdrawal leads to stimulation of prostaglandin F2α production via ROS-induced NFkappa β activation [83]. The mechanism of menstruation is unclear, and activation of the transcription factor NFkappa β may be a piece in the puzzle.

Ovarian epithelial cancer is the most common type of ovarian cancer. Ovarian epithelial inflammation has been suggested as an etiological factor in ovarian epithelial cancer [11,84]. The mechanisms that bring about follicular rupture result in the exposure of the ovarian surface epithelial cells to deleterious agents (e.g. free radicals and TNF-α) [85,86]. Thus, incessant ovulation and its complex articulation by OS, inflammation and cytokines repeated cyclically may be involved in the etiopathogenesis of ovarian cancer [86]. Factors that inhibit ovulation such as oral contraceptives reduce the risk of epithelial ovarian cancer [87,88]. Recent studies point towards a role of genes active in the process of metabolism of oxidation products, in the etiology of ovarian cancer [89].

#### 2.3 Reactive oxygen species and mediators of angiogenesis

Angiogenesis is a pathophysiological process involving formation of blood vessels from preexisting vessels. The induction of angiogenesis occurs when there is a deficiency of oxygen in tissues. This process of neovascularization results from hypoxia and induction of various angiogenic factors, and it has a role to play in physiological processes such as follicular development, endometrial growth, embryo development, growth of placental vessels and wound repair [90,91]. Angiogenesis is important for cyclical regeneration of endometrium in the menstrual cycle. A complex cytokine influence at the maternal-fetal interface creates the conditions that are necessary to support embryo implantation in the endometrium [92,93]. Any imbalance between the cytokines and angiogenesis factors could result in implantation failure and pregnancy loss [94]. Critical changes occur in the vascular system, and these changes accompany follicular growth. Follicular growth, selection of dominant follicle, corpus luteum formation, endometrial differentiation and embryo formation are key processes dependent on neovascularization [90,95]. As the endometrium grows in the menstrual cycle, vessel regeneration occurs (i.e. spiral arterioles and capillaries) [96]. Estrogens promote angiogenesis in the endometrium by controlling the expression of factors such as VEGF [97]. ROS generated from NADP (H) oxidase is critical for VEGF signaling in vitro and angiogenesis in vivo [98]. Small amounts of ROS are produced from endothelial NADP (H) oxidase activated by growth factors and cytokines.

ROS that are generated in and around the vascular endothelium may play a role in normal cellular signaling mechanisms. They may also be an important causative factors in endothelial dysfunction that leads to the development of atherosclerosis, diabetes complications and ischemia perfusion injury [98,99]. The molecular mechanism by which the oxidant status of cells modulates angiogenesis is not completely understood. As our understanding the role ROS-induced angiogenesis plays in atherosclerosis and myocardial angiogenesis grows, future studies should investigate the role ROS plays in the angiogenesis in the female reproductive tract.

#### 2.4 Reactive oxygen species and the endometrium

There is a cyclical variation in the expression of superoxide dismutase (SOD) in the endometrium. SOD activity decreases in the late secretory phase while ROS levels increase [100]. These changes have been hypothesized to be important in the genesis of menstruation and endometrial shedding. The levels of prostaglandin F2 α increase towards the late secretory phase and ROS triggers the release of prostaglandin F2 α in vitro [101]. Stimulation of the cyclooxygenase enzyme is brought about by ROS via activation of the transcription factor NFKappa β, suggesting a mechanism for menstruation [83].

#### 2.5 ROS and endometriosis

Increased generation of ROS by activated peritoneal macrophages has been reported in the peritoneal fluid [102]. Conflicting results were reported in further studies with large patient numbers, which failed to demonstrate an antioxidant or oxidant balance [74,103]. ROS levels in peritoneal fluid of patients with endometriosis were not significantly higher than controls.

An increased titer of autoantibodies related to OS has been reported in women with endometriosis resulting in an increase in serum autoantibody titers to oxidatively modified low density lipoproteins [104]. An OS-induced increase in autoantibody titers in the peritoneal fluid has been demonstrated in women with endometriosis. Elevated levels of the marker of lipid peroxidation lysophophatidyl choline, a potent chemotactic factor for monocytes/T-lymphocytes, were seen in the peritoneal fluid of women with endometriosis [105]. Non-terminal oxidation may have a role in the pathophysiology of endometriosis. Minimally oxidized low density lipoprotein (LDL) (M-LDL) is present in peritoneal fluid of women with endometriosis in place of the terminally oxidized LDL (Ox-LDL) [106]. The ratio of lysophosphatidyl choline, a breakdown product of Ox-LDL, to phosphatidyl choline suggests M-LDL rather than Ox-LDL. Modest levels of OS induced proliferation of endometrial stromal cells in vitro, was inhibited by antioxidants [107]. RU486, a potent antiprogestational agent with antioxidant activity also decreased proliferation of epithelial and stromal cells [108].

#### 2.6 Reactive oxygen species and the ovary

Markers of oxidative stress such as superoxide dismutase, Cu-Zn superoxide dismutase, Mn superoxide dismutase, glutathione peroxidase, γ glutamyl synthetase and lipid peroxides have been investigated by immunohistochemical localization, m-RNA expression and thiobarbituric acid method [4,14,41]. The expression of various biomarkers of OS has been demonstrated in normal cycling human ovaries [13,14]. All follicular stages have been examined for SOD expression including primordial, primary, preantral and nondominant antral follicles in follicular phase, dominant follicles and atretic follicles [14]. ROS may have a regulatory role in oocyte maturation, folliculogenesis, ovarian steroidogenesis and luteolysis. There is a delicate balance between ROS and antioxidant enzymes in the ovarian tissues. The antioxidant enzymes neutralize ROS production and protect the oocyte and embryo.

The presence of superoxide dismutase in the ovary, revealed intense staining by immunohistochemistry in the theca interna cells in the antral follicles [13]. Antibody to Ad4-binding protein (Ad4BP) was utilized to localize Ad4BP in the nuclei of theca and granulosa cells. Ad4BP is a steroidogenic transcription factor that induces transcription of the steroidogenic P450 enzyme. Thus, it controls steroidogenesis in the ovaries. The correlation between Ad4BP and superoxide dismutase expression suggests an association between OS and ovarian steroidogeneis [14].

Both human granulosa and luteal cells respond to hydrogen peroxide with an extirpation of gonadotropin action and inhibition of progesterone secretion [11]. The production of both progesterone and estradiol hormones is reduced when hydrogen peroxide is added to a culture of human chorionic gonadotropin-stimulated luteal cells. Hydrogen peroxide lowers both cAMP dependent and non-cAMP dependent steroidogenesis [109]. The role of hCG (human chorionic gonadotropin) in the expression of the antioxidant enzyme superoxide dismutase (SOD) has been investigated. Corpora lutea collected from patients at hysterectomy and surgery for ectopic pregnancy were studied [14]. The Cu-Zn SOD expression in the corpora lutea paralleled levels of progesterone and these levels rose from early to mid luteal phase and decreased during the regression of the corpus luteum. However, in the corpus luteum from pregnant patients, the mRNA expression for Cu-Zn superoxide dismutase was significantly higher than that in midcycle corpora lutea. This enhanced expression of luteal Cu-Zn SOD may be due to hCG and hCG may have an important role in maintenance of corpus luteal function in pregnancy.

Levels of three oxidative stress biomarkers, conjugated dienes, lipid hydroperoxide and thiobarabituric acid were determined in preovulatory follicles. Concentration gradient was found to exist as levels of all three markers were significantly lower in the follicular fluid compared with serum levels [15]. The preovulatory follicle has a potent antioxidant defense, which is depleted by the intense peroxidation [15]. Glutathione peroxidase may also maintain low levels of hydroperoxides inside the follicle and thus play an important role in gametogenesis and fertilization [42].

#### 2.7 Nitric Oxide synthase in female reproduction

The production of a viable oocyte is modulated by a complex interaction of endocrine, paracrine and autocrine factors leading to follicular maturation, granulosa cell maturation, ovulation and luteinization. Many hormonal and paracrine factors determine oocyte competence and embryo quality. Steroid hormones and local autocrine and paracrine factors influence ovarian stromal cells. The gonadotropins act through complex, multiple local signaling pathways. Cyclic AMP is thought to be the second messenger to bring about the effect of luteinizing hormone and follicular stimulating hormone [110]. In turn, cyclic AMP may activate other signaling pathways. Cyclic guanosine monophosphate (cGMP)-a cyclic nucleotide has also been proposed as a second messenger pathway. The effects of NO are proposed to be mediated through cGMP as a second messenger or by generation of ROS resulting from interaction of NO with superoxide radicals [111].

NO generated by macrophages in response to invading microbes acts as an antimicrobial agent [21]. Neurons, blood vessels and cells of the immune system are integral parts of the reproductive organs, and in view of the important functional role that NO plays in those systems, it is highly likely that NO is an important regulator of the biology and physiology of the reproductive system. NO has established itself as a polyvalent molecule that plays a decisive role in regulating multiple functions within the female as well as male reproductive system [21]. As a final immune effector, NO generated by inducible NO synthase, kills pathogens and abnormal cells but may play a detrimental role by damaging normal host tissue and cells, especially when inducible NO synthase is persistently expressed [20].

#### 2.8 Nitric oxide synthase and fallopian tubes

The presence of NO synthase enzymes, both the constitutive and the inducible forms was delineated by immunhistochemistry and the presence of NADPH-diaphorase activity in human tubal cells [112,113]. The production of NO was demonstrated by positive NADPH diaphorase activity in the human fallopian tube. An endogenous NO system exists in the fallopian tubes [114]. NO has a relaxing effect on smooth muscles and it has similar effects on tubular contractility. Deficiency of NO may lead to tubal motility dysfunction, resulting in retention of the ovum, delayed sperm transport and infertility. Infertility associated with urogenital tract infections is associated with diminished sperm motility and viability. Increased NO levels in the fallopian tubes are cytotoxic to the invading microbes and also may be toxic to spermatozoa [114].

#### 2.9 Nitric oxide synthase, endometrium, and myometrium

Expression of endothelial and inducible NO synthase have been demonstrated in the human endometrium [115], and the endometrial vessels [116]. Endothelial NO synthase, originally identified in vascular endothelial cells, is distributed in glandular surface epithelial cells in the human endometrium. NO also regulates the microvasculature of the endometrium and is important in menstruation.

Endothelial NOS like immunoreactivity has been reported in the endothelial cells lining the vessels, endometrium and endometrial glandular epithelial cells and myometrium [117]. Inducible NOS like immunoreactivity was detected in decidualised stromal cells and also expressed in tissues from first trimester of pregnancy. Thus, NO has a role to play in decidualisation of the endometrium and preparation of the endometrium for implantation.

Highest levels of expression of endothelial NOS mRNA have been reported in the late secretory phase of the endometrium [115]. In addition, NO might participate in the regulation of uterine contraction [118]. In normal fertile woman, the contractions increase throughout the proliferative and periovulatory phases, and decrease in the secretory phase. From this point, NO is synergistic with progesterone and might relax uterine contraction in the secretory phase in a paracrine fashion. Nitric oxide regulates the endometrial, myometrial and microvascular functions in the uterus by paracrine functions. The effects of the antiprogestational agent, Mifepristone on the endothelial NOS expression in the endometrium were investigated during the implantation phase [119]. Mifepristone inhibited the endometrial glandular epithelial eNOS expression and did not affect the endothelilal eNOS. The results supported the role of epithelial eNOS in endometrial receptivity in the perimplantation period. Significant increase in the levels of serum metabolites of NOS amongst users of progestin only contraceptives has been reported [120]. A positive correlation was also demonstrated between NO levels and prolonged/heavy bleeding. Thus NO may be involved in the pathophysiology of menorrhagia.

NO plays a significant role in pregnancy and labor. Expression of inducible NOS was highest in patients with preterm pregnancy and not in patients in term labor. The expression of these enzymes decreased by 75% at term and was barely detectable in preterm in labor patients or term labor patients [121] reiterating that NO has a role in maintenance of uterine quiescence. However other authors have reported conflicting results. Induction of iNOS expression was demonstrated in fetal membranes in labour and in in-vitro studies [122]. Higher NO metabolite concentrations were demonstrated in amniotic fluid collected from women in labor than in non-laboring patients, both at term (15.4 ± 1.6 vs. 6.8 ± 0.6 microM/mg creatinine) and preterm (16.7 ± 2.0 vs. 7.0 ± 0.8 microM/mg creatinine) [123].

#### 2.10 Nitric oxide synthase and endometriosis

Endometriosis is found in about 35% of infertile women who have laparoscopy as part of their infertility workup [71]. Production of ROS by peritoneal fluid mononuclear cells was reported to be associated with endometriosis [75]. Low levels of NO are important in ovarian function and implantation and cause relaxation of oviduct musculature [112]. High levels of NO are reported as having deleterious effects on sperm motility, toxic to embryos and inhibit implantation [124,125]. In vitro fertilization, a process that avoids contact of gametes and embryos with potentially toxic peritoneal and oviductal factors associated with endometriosis (e.g., NOS, ROS) improves the chances of conception in these women. NO is a free radical with deleterious effects and is an important bioregulator of apoptosis [126]. Activation of polymorphonuclear leucocytes and macrophages leads to increased production of ROS [102]. Increase in number and activity of macrophages is accompanied by release of more cytokines and other immune mediators, such as NO. This was initially implicated in low-grade inflammation, while elevated peritoneal NO is consistent with the increased number and activity of macrophages [20]. High levels of NO, such as those produced by macrophages, can negatively influence fertility in several ways. Changes in peritoneal fluid, an environment that hosts the process of ovulation, gamete transportation, sperm oocyte interaction, fertilization, and early embryonic development, might affect all these steps of reproduction [2,20,127]. Studies investigating the association of nitric oxide levels and lipid peroxides and reactive oxygen species in peritoneal fluid did not find any significant difference in patients with or without endometriosis [103,128,129] Conflicting results were obtained in studies conducted by Szczepanska et al [2]. The total antioxidant capacity reduced and the individual antioxidant enzymes like superoxide dismutase were significantly lower in the peritoneal fluid of women with endometriosis-associated infertility. The lipid peroxide levels were the highest amongst patients with endometriosis suggesting role of ROS in the development of the disease process [2]. There is a cyclical expression of mRNA of NOS in the epithelial glands of the human endometrium. Higher amounts of NO and NOS are seen in the endometrium of women with endometriosis [28,130,131]. NOS expression in the ectopic endometrium of patients with adenomyosis is continuous throughout the menstrual cycle [132].

Peritoneal fluid NO levels, peritoneal macrophage NOS activity, and peritoneal macrophage inducible NOS protein expression has been examined in women with endometriosis-associated infertility. Peritoneal macrophages express higher levels of NOS, have higher NOS enzyme activity, and produce more NO in response to immune stimulation in vitro [23]. High levels of NO adversely affect sperm, embryos, implantation, and oviductal function, indicating that reduction in the peritoneal fluid NO production or blocking NO effects may improve fertility in women with endometriosis [23]. However, generation of peroxynitrite by ectopic endometrium has been reported in patients with adenomyosis. Expressions of endothelial and inducible NO synthase and peroxynitrite generation was markedly reduced after GnRH agonist therapy, supporting their potential role in the pathophysiology of adenomyosis [132]. Serum NO levels are suppressed by GnRH agonist and upregulated by gonadotropin stimulation during controlled ovarian stimulation in female partners from couples with male factor infertility [133]. Maximal levels were measured at the time of ovulation in the same study. Elevated NO production was not demonstrated in patients with ovarian hyperstimulation.

Increased levels of NO were demonstrated in the peritoneal fluid of patients with endometriosis [20,23]. It has also been hypothesized that ROS may have a role in formation of adhesions associated with endometriosis [134]. Patients with endometriosis show a higher 8-hydroxy 1-deoxyguanosine index compared to patients with other causes of infertility, such as tubal, male factor or idiopathic causes [52].

Expression of NOS is elevated in patients with endometriosis, and a common polymorphism of exon 7 at nucleotide 894 in the endothelial NOS gene may be associated with endometriosis [135]. Hence variations in the expression of the eNOS gene may be involved in endometrial angiogenesis and thus modulate the process of endometriosis.

Expression of endothelial NO synthase in the endometrium of patients with endometriosis or adenomyosis is persistently marked throughout the menstrual cycle [132]. Many investigators have reported increased expression of endothelial NOS in the glandular endometrium in patients with endometriosis [28,130]. Inducible NOS isoform is elevated in tissues of patients with endometriosis [131]. The endometrial development affects embryo implantation, and inconsistent development between endometrium and embryo could impede embryo implantation. Nitric oxide affects fecundity in endometriosis and adenomyosis [136]. Significant differences are seen in the uterine hyperperistalsis and dysperistalsis in patients with endometriosis compared with the control groups, and this may be responsible for disturbed sperm transport and reduced fertility [137].

Various cytokines secreted from endometrial cells, immune cells, or macrophages stimulate endothelial NO synthase to release NO [3,28,136]. These abnormal immune responses might eventually stimulate macrophages and/or endometrial cells to persistently produce a large amount of NO and inhibit implantation [138]. Increased expression of endothelial NO synthase has been reported throughout the menstrual cycle in the endometrium of women with endometriosis [139].

#### 2.11 Nitric oxide synthase and the ovary

Ovarian follicullogenesis not only involves gonadotropins and the steroids, but it also involves local autocrine and paracrine factors. Nitric oxide radical is one of the local factors involved in ovarian follicullogeneis and steroidogenesis. Nitric oxide acts through activation of various iron containing enzymes. It binds to the heme containing enzyme guanylate cyclase, which activates the cyclic nucleotide cyclic-GMP [110]. Plasma concentrations of nitrate monitored during the follicular cycle, have revealed peak levels at ovulation [133,140]. Nitric oxide inhibits ovarian steroidogenesis [52]. The presence of endothelial NO synthase in human corpora lutea and the expression has been reported in the mid and early luteal phase and to a lesser extent in the late luteal [53]. Nitric oxide inhibits steroidogenesis in the corpus luteum and has luteolytic action mediated through increased prostaglandins and by apoptosis [53,141].

Follicular fluid NO seems to be produced by either endothelial NO synthase or induced NO synthase. However, in the normal physiological conditions follicular fluid NO seems to be synthesized from granulosa cells by endothelial NO synthase, since in isolated human follicular cells at least 90% of cells are granulosa cells even though macrophages and lymphocytes are present as well. In patients undergoing IVF, a positive correlation was determined between follicular fluid nitrate/nitrite levels and the follicular volume as well as the serum estradiol concentration [142]. In contrast to these findings, Manau et al, found no association between follicular fluid nitrite/nitrate levels and parameters of ovarian response [143]. Biomarkers like serum nitric oxide measurements cannot be used as predicting success with IVF [143,144]. Serum nitrate concentration may not be a good biomarker because of the short half-life of NO. Follicular blood flow was found to be a better prognostic factor for predicting successful outcomes with IVF than follicular NO levels [138]. Follicular fluid NO levels were altered in patients with infertility associated diseases. NO follicular fluid levels were significantly higher in patients with endometriosis or hydrosalpinx compared to patients with tubal obstruction [29]. No correlation was reported between the follicular NO levels and follicle maturity or follicle quality.

Some studies have demonstrated the relationship between NO concentrations in follicular growth and programmed follicular cell death (apoptosis). Folliculogenesis involves the participation of both growth of the follicle and apoptosis. Nitric oxide regulates both of these processes [21]. Sugino et al studied the role of nitric oxide in follicular atresia and apoptosis, in patients undergoing IVF and found that the smaller follicles had significantly elevated percentage of apoptotic granulosa cells with nuclear fragmentation [58]. Low concentrations of NO may prevent apoptosis, however pathologically high concentrations of NO, as well as increased superoxide generation by NO synthase due to lack of arginine, may promote cell death by peroxynitrite generation [21]. Nitric oxide involvement in various ovarian functions has been suggested. The presence of NO in the follicular fluid and the expression of NO synthase in follicles and corpus luteum have been reported [19,141,143,145].

Plasma concentration of NO was shown to increase in the follicular phase compared with the secretory phase and peaked at midcycle [140]. Nitric oxide elicited a positive effect on women with poor ovarian response compared to controlled ovarian stimulation [146]. Upregulated NO is harmful to implantation and pregnancy among patients with tubal factor infertility after controlled ovarian stimulation [147]. Serum NO levels were elevated amongst nonpregnant patients with tubal or peritoneal factor infertility [124].

Follicular fluid NO level is not associated with maturity or quality of oocyte and no significant differences were seen in concentrations of NO of follicular fluid among large, medium, or small follicle size. Higher TNF-α concentrations in follicular fluid correlated with poor oocyte quality [29]. Whereas, follicular fluid nitrite or nitrate levels were significantly lower in follicles containing mature oocytes that fertilized compared with those that did not [148]. Follicular NO has been reported to correlate negatively with embryo quality and the rate of embryo cleavage [124,147,148]. The beneficial effects of NO donors in patients with intrauterine growth retardation (IUGR) and inhibition of pre-term labor has been studied [149,150]. Using a nitroglycerine (NTG) patch, which is a NO donor, did not significantly affect the final outcome in patients undergoing in-vitro fertilization. In addition, neither placebo nor the nitroglycerine patch improved the flow resistance in the uterine artery [22]. NO donors and elevated serum NO was associated with implantation failure resulting in decreased fertility [138].

### 3. Assisted reproduction

Assisted reproductive technique (ART) involves the direct manipulation of the human oocytes, sperm or embryos outside the body, to establish a pregnancy. A variety of causative factors of infertility can be indications for ART, i.e. tubal factor, endometriosis, male factor and unexplained infertility [151,152]. Assisted reproductive techniques offer excellent opportunities to infertile couples for achieving pregnancy. There may be multiple sources of ROS in an IVF setting including the oocytes, cumulus cell mass, or spermatozoa used for insemination [153].

#### 3.1 Oxidative stress and its impact on ART

The follicular fluid microenvironment has a crucial role in determining the quality of the oocyte. This in turn impacts the fertilization rate and the embryo quality. Oxidative stress markers have been localized in the follicular fluid in patients undergoing IVF/embryo transfer (ET) [4,51,154,155]. Low intrafollicular oxygenation has been associated with decreased oocyte developmental potential as reflected by increasing frequency of oocyte cytoplasmic defects, impaired cleavage and abnormal chromosomal segregation in oocytes from poorly vascularised follicles [156]. ROS may be responsible for causing increased embryo fragmentation, resulting from increasing apoptosis [157]. Thus increasing ROS levels are not conducive to embryo growth and result in impaired development. Current studies are focusing on the ability of growth factors to protect in vitro cultured embryos from the detrimental effects of ROS such as apoptosis. These growth factors are normally found in the fallopian tubes and endometrium. The factors being investigated are: Insulin growth factor (IGF)-1, and Epidermal growth factor (EGF) in mouse embryos, which in many respects are similar to human embryos [158].

Exogenous gonadotropin has a stimulatory effect on the follicular content of iron, which is a potent oxidant, catalyses generation of free radicals in Haber-Weiss reaction. Iron overload in thalassemia acts as a redox-active center and there is resultant increase in the production of free radicals [159]. Increase in free radicals was reported in follicular fluid of patients with thalassemia. The spectrum of initial hypogonadism and later gonadal failure in thalassemia, results from the injury mediated by free radicals.

Increase in TAC was seen in follicular fluid of oocytes that later were successfully fertilized. Therefore, lower total antioxidant capacity is predictive of decreased fertilization potential [154]. Lower levels were associated with increased viability of the embryos until the time of transfer, and the fertilization potential decreased with decreasing concentrations of total antioxidants. Similarly mean glutathione peroxidase levels were increased, in follicles yielding oocytes that were subsequently fertilized [42]. Levels of ROS were reported to be significantly lower in patients who did not become pregnant compared with those who became pregnant [4]. Thus intrafollicular ROS levels may be used as a potential marker for predicting success with IVF. Studies determining normal TAC levels of the follicular fluid in unstimulated cycles are lacking.

In addition levels of selenium in follicular fluid of women with unexplained infertility were lower than those in women with tubal factor or male factor infertility [42]. Higher levels of superoxide dismutase activity were present in fluid from follicles whose oocytes did not fertilize compared with those that did [12]. These discrepancies may be due to the fact that the studies measured different parameters. The effects of follicular OS on oocyte maturation, fertilization and pregnancy have also been studied [51]. Patients who became pregnant following IVF or ICSI had higher lipid peroxidation levels and TAC. Both markers were unable to predict embryo quality. Pregnancy rates and levels of lipid peroxidation and TAC demonstrated a positive correlation.

OS in follicular fluid from women undergoing IVF was inversely correlated with the women's age [160]. Using a thermochemiluminescence assay, the slope was found to positively correlate with maximal serum estradiol levels, number of mature oocytes and number of cleaved embryos and inversely with the number of gonadotropin ampoules used. The pregnancy rate achieved was 28% and all pregnancies occurred when the thermochemiluminescence amplitude was small. This is in agreement with another study that reported minimal levels of OS were necessary for achieving pregnancy [51]. Follicular fluid ROS and lipid peroxidation levels may be markers for success with IVF.

Oocyte quality is a very important determining factor in the outcome of IVF/ET. 8-hydroxy-2-deoxyguanosine is a reliable indicator of DNA damage caused by oxidative stress. This compound is an indicator of OS in various other disease processes i.e. renal carcinogenesis, and diabetes mellitus. Higher levels of 8 hydroxy 2-deoxyguanosine were associated with lower fertilization rates and poor embryo quality [52]. Higher levels of 8-hydroxy 2-deoxyguanosine are also seen in granulosa cells of patients with endometriosis, and this may impair the quality of oocytes.

Other OS markers such as thiobarbituric acid-reactive substances, conjugated dienes and lipid hydroperoxides have been studied in the preovulatory follicular fluid [15]. No correlation was seen between these markers and IVF outcome (fertilization rates or biochemical pregnancies) [15]. A potent antioxidant system may be present in the follicular fluid as indicated by low levels of all 3 biomarkers of oxidative stress in the follicular fluid. A recent chemiluminescence study examined the follicular fluid obtained from patients undergoing IVF. Hydrogen peroxide was utilized for the induction of chemiluminescence. The study found that a delicate balance was maintained by pro-oxidant/antioxidants in the follicular fluid [161].

Smoking has been associated with prolonged and dose-dependent adverse effects on ovarian function [162]. According to a meta-analysis, the overall value of the odds ratio for the risk of infertility associated with smoking was 1.60 [95% confidence interval (CI) 1.34–1.91]. ARTs, including IVF, are further shedding light on the effects smoking has on follicular health. Intrafollicular exposure to cotinine increases lipid peroxidation in the follicle [155]. Carotenoids have gained attention because they are similar to vitamin E in that they are very potent antioxidants and react with ROS; the presence of carotenoids has been demonstrated in the follicular fluid [163]. Concentrations of carotenoids, retinol and alpha tocopherol were found to be significantly higher in follicular fluid than in plasma.

Melatonin was investigated as a drug to improve oocyte quality in patients failing to get pregnant in earlier IVF cycles because of poor quality oocytes [164]. A significant reduction in the number of degenerate oocytes was reported, and the number of fertilized embryos increased. Increased follicular concentrations of melatonin reduced lipid peroxide concentration and may have prevented DNA damage.

#### 3.2 Redox and early embryo development

Physiological levels of redox may be important for embryogenesis. Overproduction of ROS is detrimental for the embryo, resulting from impaired intracellular milieu and disturbed metabolism [17,165]. Superoxide anion, hydrogen peroxide and hydroxyl radical, can have detrimental effects on the fetus. Oxidative stress can be generated by spermatozoa, leucocytes, and by events such as sperm mediated oocyte activation and activation of the embryonic genome [165]. The ROS generation can result from oxidative phosphorylation occurring in the mitochondria. Electrons leak from the electron transport chain at the inner mitochondrial membranes. These electrons are transferred to the oxygen molecule, resulting in an unpaired electron in the orbit. This leads to the generation of the superoxide molecule. The other points of generation of ROS are the cytoplasmic NADPH-oxidase, cytochrome p450 enzymes and the xanthine oxidoreductase enzymes. Excessive OS can have deleterious effects on the cellular milieu and can result in impaired cellular growth in the embryo or apoptosis resulting in embryo fragmentation. Thus OS mediated damage of macromolecules plays a role in fetal embryopathies. Deficient folate levels in the mother result in elevated homocysteine levels. Homocysteine induced oxidative stress has been proposed as a potential factor for causing apoptosis and disrupting palate development and causing cleft palate [166]. Oxidative stress mediated damage of the macromolecules has been proposed as a mechanism of thalidomide induced embryopathy and other embryopathies [167,168].

Hyperglycemia/diabetes induced down regulation of cycloxygenase-2 gene expression in the embryo results in low PGE_2 _levels and diabetic embryopathy [169]. The protective role of the enzyme G6PD (Glucose 6-phophate dehydrogenase) against oxidative stress has been demonstrated in an animal study and embryopathies were prevented by protecting the embryos against oxidative stress [170].

#### 3.3 Effect of oxygen concentration on in-vitro embryo development

Early embryo development in mammals occurs from fertilization through differentiation of principal organ systems in a low oxygen environment [168]. A marginal improvement in preimplantation embryonic viability has been reported under low oxygen concentrations in patients undergoing IVF and ICSI [171]. Lower concentrations of oxygen in in-vitro culture of porcine embryos decreased the H_2_O_2 _content and resulted in reduced DNA fragmentation, which thereby improved developmental ability [172]. The higher oxygen concentration of 20% have been associated with lower developmental competence. Accelerated development was seen under low (5%) oxygen concentrations.

#### 3.4 Role of ROS in IVF media

ROS may be generated endogenously or exogenously, but either way it can affect the oocyte and embryo. IVF culture media may be the exogenous site of ROS generation affecting the oocytes and the preimplantation embryo. There are some specific events in embryo development associated with a change in the redox state. It has been suggested that redox may have a causative role in sperm mediated oocyte activation, embryonic genome activation and embryonic hatching from the zona pellucida [165]. Higher day 1 ROS levels in culture media were associated with delayed embryonic development, high fragmentation and development of morphologically abnormal blastocysts after prolonged culture. A significant correlation was reported between increased ROS levels in Day1 culture media and lower fertilization rates in patients undergoing ICSI [153]. Lower ROS levels were associated with higher fertilization rates, indicating the physiological relevance of low levels of ROS.

Incubation of poor quality embryos was associated with a decline in TAC in the preimplantation embryo culture medium after 24 hours incubation. Poor quality embryos may be associated with increased generation of ROS [173]. HEPES [(2-hydroxyethyl) piperazine-1-ethanesulfonic acid] was found to be the most potent protector compared to human tubal fluid media and polyvinyl alcohol against DNA damage occurring in spermatozoa, as determined by plasmid relaxation assay which measures the plasmid DNA damage [174]. IVF media supplemented with human serum albumin (10 mgm/mL), glucose (2.78 Mmol), 1% polyvinyl alcohol, 5% polyvinylpyrrolidone, sucrose (100 mM), 60% Percoll, human tubal fluid, human tubal fluid media, catalase (1 and 10 IU), and HEPES (21 mMol) scavenge ROS and confer protection from DNA damage [174].

#### 3.5 Strategies to overcome oxidative stress in infertility/ART

Considerable interest has been generated in the use of antioxidants to overcome the adverse and pathological results of OS. Oxidative stress leads to luteal regression, [43] resulting in a lack of luteal support to a pregnancy [33]. OS can damage oocytes in developing follicles, oocytes and spermatozoa in the peritoneal cavity, or embryo in fallopian tube [17,153] or through redox (pro-oxidant and antioxidant) imbalance. OS can be overcome by reducing generation of ROS or increasing the amounts of antioxidants available. The literature contains studies that used nutritional supplements and antioxidants like vitamin C supplementation to protect against ROS and OS. However, there is lack of consensus on the type and dosage of antioxidants to be used. Clinical evidence on the benefits of antioxidant supplementation is equivocal.

Current evidence supports the use of systemic antioxidants for management of selected cases of male infertility [175]. Randomised controlled trials investigating antioxidants in female infertility are few and lack power because of the small patient numbers. In a recent randomized controlled, multi-center study, the effect of vitamin C supplementation (750 mg/day) in patients with a luteal phase defect was reported; pregnancy rates were higher in the treatment group than in the controls [176]. Similarly, concentrations of antioxidants were found to be significantly lower in women with a history of recurrent miscarriages and luteal phase defects than in healthy women [177]. Vitamin C concentrations were higher in the follicular fluid of patients supplemented with vitamin C than that of the controls. Pregnancy rate was higher in the supplemented group than in the control group although the difference was not statistically significant [178].

In a double blinded, placebo-controlled pilot study, the impact of a nutritional supplement containing vitamin E, iron, zinc, selenium, L-arginine was examined [179]. The mean mid-luteal progesterone levels increased from 8.2 ng/mL to 12.8 ng/mL, (p = 0.08), and the patients had a significant increase in ovulation and pregnancy rates (33% pregnant, p < 0.01) [179]. In a study looking at short-term supplementation with high doses of ascorbic acid during the luteal phase in IVF, the clinical pregnancy rate and implantation rate did not improve [180]. There is lack of consensus on antioxidant supplementation in idiopathic infertility and randomized controlled trials need to be designed with sufficient power and patient numbers to investigate this issue.

Fertilization and embryo development in vivo occurs in an environment of low oxygen tension [168]. During ART, it is important to avoid conditions that promote ROS generation and expose gametes and embryos to ROS. During culture, low oxygen tension is more effective at improving the implantation and pregnancy rate than higher oxygen tension [181]. Similarly, higher implantation and clinical pregnancy rates are reported when antioxidant supplemented media is used rather than standard media without antioxidants. Metal ions can sometimes result in the production of oxidants. Metal ions can also increase the production of ROS directly through the Haber-Weiss reaction. It may be useful to add metal ion chelating agents to the culture media to decrease the production of oxidants [181].

Amino acids added to the IVF media also have antioxidant properties. Adding ascorbate during cryopreservation reduces hydrogen peroxide levels and thus the oxidative distress in mammalian embryos [182]. As a consequence, the embryo development improved with enhanced blastocyst development rates. A significant negative association has been reported between duration of smoking and fertilization rates in IVF procedures. Eliminating the smoking factor would help improve fertility and ART outcomes [178]. Because a history of smoking is associated with high concentrations of OS, in-vivo antioxidants can be recommended in infertile women who smoke [155].

Follicular vascularity determines the intrafollicular oxygen content and the developmental potential of the oocyte [156,183]. Intrafollicular hypoxia results in chromosomal segregation disorders and deleterious mosaicisms in the embryo. Sildenafil, an inhibitor of phosphodiesterase enzyme, prevents the breakdown of cGMP and potentiates the effects of NO on vascular smooth muscle. Vaginal Sildenafil and L-arginine have been investigated for their potential to improve intrafollicular blood flow by potentiating the actions of NO on vascular smooth muscle. It augments the effect of NO in inducing vasodilatation and thus improving uterine blood flow. A recent study reported that Sildenafil, administered on day 3 of the menstrual cycle, appeared effective in improving uterine artery blood flow and endometrial development [184]. The same group in a subsequent cohort of 105 patients with infertility and previous failures at IVF were able to achieve higher implantation and pregnancy rates with vaginal Sildenafil [185]

Oral L-arginine supplementation in poor responder patients, during controlled ovarian stimulation may improve ovarian response, endometrial receptivity and pregnancy rate by increasing the flow around the follicles, and uterine flow [146]. Follicular fluid concentrations of nitrite/nitrate inversely correlated with embryo quality. Although the embryo quality was poor, L-arginine supplementation in normally responding patients resulted in higher follicular fluid arginine levels compared to the poor responders and increased follicular recruitment [147]. NO derivatives in higher doses in follicular fluid may cause cytostatic and cytotoxic effects and may have detrimental consequences on embryo quality, implantation and pregnancy rate.

Mechanical removal of ROS in IVF/ET has been examined [186]. Cumulus oophorus rinsing is performed to overcome the deleterious effects of ROS in patients with ovarian endometriosis [186]. ROS has deleterious effects on both the oocyte and the embryo quality. The deleterious effects of TNF-α cytokines and reactive oxygen species, which were increased in the peritoneal fluid of patients with endometriosis and unexplained infertility, were prevented, by the rinsing procedure.

#### 3.6 Critical review of OS, ovary and ART

A comprehensive review of the published literature reveals that the role of oxidative stress is controversial due to the differences in the nature of materials examined, (i.e. follicular fluid, embryos, and culture mediums). We can conclude the number of articles on oxidative stress in the last 5 years have significantly increased compared to the previous 5 years indicating that more studies are being conducted to understand the role of oxidative stress in female reproduction. The effects of ROS studied and its ability to influence female reproduction have been studied on various endpoints in terms of the oocyte, fertilization, embryo and pregnancy. Different markers of oxidative stress are reported in various studies and the sensitivity and specifity of the various biomarkers are not known. While some research is focused on studying the antioxidant capacity others focus on studying and determining the levels of oxidative stress markers. Also, there has been an assumption in the studies measuring the amount and type of antioxidants that there is an inverse correlation between oxidative stress markers and antioxidants. These studies have also variations because some have measured the total antioxidant capacity and some have measured individual enzymes like superoxide dismutase. Further studies need to be designed to validate the results of the earlier studies, with elimination of various factors leading to bias. Eliminating the bias will make the comparison of different studies acceptable and provide support to the evidence based approach. The biomarkers of oxidative stress that are studied should be similar across different studies to make the results comparable. Prospective, randomized controlled trials with stringent inclusion criteria are needed to determine the effects of antioxidants in overcoming redox in infertility patients.

### 4. Age related fertility decline, Menopause and ROS

There is an age related decline in the number and quality of follicles in females. ROS may damage the oocytes [187]. The age related decline in oocyte quality also results in increased incidence of congenital anomalies in children. The ageing of the oocytes affects many biochemical pathways which have a deleterious effect on pre- and post implantation development of the embryo [188]. The pre- and postovulatory ageing of the oocytes have also been associated with congenital anomalies, behavorial alterations, and learning disabilities in later life and constitutional diseases such as diabetes mellitus, and schizophrenia. Oxidative stress occurs at menopause because of loss of estrogens, which have antioxidant effect on low-density lipoproteins. Estrogens confer cardioprotection by lowering protein oxidation and antioxidant properties [189]. Diminished antioxidant defense is associated with osteoporosis in post-menopause. Modulation of the estrogen receptors α and β has been reported to be effected in vitro by oxidative stress [190].

### 5. Oxidative stress and pregnancy

#### 5.1 Oxidative stress and pre-eclampsia

Pre-eclampsia is associated with severe maternal and fetal morbidity and mortality [191]. Overall pre-eclampsia complicates 5% of all pregnancies and 11% of all first pregnancies. Recent evidence suggests the role of oxidative stress in pre-eclampsia. There is a reduced antioxidant response inpatients with pre-eclampsia [192,193] and reduced levels of antioxidant nutrients [194] and increased lipid peroxidation [45,194].

#### 5.2 Placental oxidative stress and Pre-eclampsia

Incomplete trophoblast invasion leads to failure of conversion of thick walled tortous spiral arteries to low resistance flaccid sinusoidal vessels [195,59]. The incomplete invasion results in impaired placental perfusion. The hypoxia/reperfusion injury leads to increased expression of xanthine oxidase and NADP (H) oxidase and resultant increased generation of superoxide anion. The increased generation of pro-oxidants tilts the balance in favor of oxidative stress, which results in increased lipid peroxidation. Biomarkers of lipid peroxidation are elevated in the placenta [45,60].

#### 5.3 Interventions to overcome oxidative stress in pre-eclampsia

There is currently no accepted method of prevention of pre-eclampsia. Antioxidants vitamin C and vitamin E have been studied in some trials for preventing pre-eclampsia. Early intervention at 16–22 weeks of pregnancy with supplementation of vitamin E and C resulted in significant reduction of pre-eclampsia in the supplemented group [196]. Supplementation in women with established pre-eclampsia did not result in any benefit [197]. Recent report of a randomized trial failed to find beneficial effects of vitamin C and E supplementation in preventing preeclampsia [198].

#### 5.4 Redox and miscarriage

Human placenta is classified as hemomonochorial. Maternal blood directly bathes the fetal trophoblast. Establishment of the maternal placental circulation is influenced by the trophoblastic invasion. Extravillous trophoblastic invasion transforms the small caliber high resistance spiral arteries into large caliber, low resistance, and high capacitance uteroplacental arteries. Abnormal placentation has been implicated in the pathogenesis of pre-eclampsia and miscarriage [199]. Pre-eclampsia is unique to human species and miscarriage is very rare in other species [200]. Abnormal placentation leads to placental oxidative stress with resultant detrimental effects on the syncitiotrophoblast and it has been proposed as a mechanism involved in the etiopathogenesis of abortion. A sharp peak in the expression of the markers of oxidative stress in the trophoblast was detected in normal pregnancies and this oxidative burst if excessive was speculated to be a cause of early pregnancy loss [168].

The etiology of recurrent pregnancy loss remains unclear and is a scientific challenge. Oxidative stress may have a role in the etiology of recurrent pregnancy loss with no known etiology. Glutathione and glutathione transferase family of enzymes have been investigated in patients who experience recurrent abortions [201,202]. Glutathione and glutathione peroxidase are both antioxidants that neutralize the free radicals and lipid peroxides to maintain the intracellular homeostasis and redox balance.

The etiology of recurrent pregnancy loss is multifactorial and involves genetic and environmental factors [203]. In a large case controlled study, gene polymorphisms of enzymes of the glutathione family, glutathione S-transferase class mu (GSTM1) were studied. Elevated risk of recurrent pregnancy loss was found to be associated with the GSTM1 genotype null polymorphism, in patients with recurrent pregnancy loss. Elevated glutathione levels in pregnant patients with history of recurrent pregnancy loss were associated with poor outcomes (i.e. abortion) [201].

#### 5.5 Term labor and the role of oxidative stress

There is increased generation of free radicals superoxide and nitric oxide in pregnancy, which results in oxidative stress [35]. Term labor induces increased lipid peroxidation, as evidenced by increased levels of the biomarker, malondialdehyde [37]. In a case controlled study, the serum levels of hydroperoxides were higher in patients in labor, compared to the controls, who were not in labor [36]. Term labor was demonstrated to cause an up regulation of the antioxidant reserve in the fetal compartment [66]. The role of oxidative stress in initiation of labor is not known.

F2-isoprostanes, reliable biomarkers of oxidative stress were shown to be significantly elevated in plasma of neonates compared to adults [204]. The study also demonstrated an inverse correlation between gestational age and plasma isoprostane levels.

#### 5.6 Interventions to overcome oxidative stress during pregnancy

Based on the understanding of the pathophysiological role of NO in the female reproductive tract, NO donors have been studied for cervical ripening at term. In a randomized controlled study, vaginal administration of isosorbide dinitrate induced cervical ripening at term [205]. Oxidative stress leads to focal collagen damage in the fetal membranes and result in preterm labor [39,206]. Antioxidant supplementation has been investigated in preterm labor and pre-eclampsia for beneficial effects [196,207]. Another randomized double-blinded placebo controlled trial initiated in 2003 will examine women with type-1 diabetes. These women were randomized to receive antioxidant supplementation with vitamin C and vitamin E. Benefits of antioxidant supplementation will be investigated in patients with type-1 diabetes and the incidence of pre-eclampsia in this group of patients will be studied [208]. In addition the secondary outcomes of birth weight centile and the endothelial activation indicated by PAI-1/PAI-2 (plasminogen activator-1/plasminogen activator-2) ratio will also be studied [208].

### 6. Future perspectives in antioxidant therapy

Antioxidants prevent the actions of the free radicals of oxidizing the substrate. Studies conducted in humans aimed at delineating the association of TAC content of food with incidence of chronic diseases [209]. The nutrients that are being studied for their effects on chronic diseases are vitamin C, Vitamin E, carotenoids and selenium. Pregnant women with HIV infection, selenium deficiency or micronutrient deficiencies like vitamin C and vitamin A, were found to have adverse clinical outcomes in large prospective studies [210,211]. There is increasing argument for increasing the selenium intake in these patients. There is emerging enthusiasm in the use of antioxidants, natural or synthetic. Small molecules that mimic antioxidant enzymes are the new tools being developed in the antioxidant armamentarium [212]. These are cell membrane permeable unlike the natural superoxide dismutase. Antioxidants targeting cellular organelles like mitochondria are also being investigated. Gene polymorphisms of the glutathione S-transferase family and myeloperoxides and their association with endometriosis, is an area of recent interest, which is promising [26].

## 7. Conclusion

The literature provides some evidence of oxidative stress influencing the entire reproductive span of a woman, even the menopausal years. OS plays a role in multiple physiological processes from oocyte maturation to fertilization and embryo development. There is burgeoning literature on the involvement of OS in the pathoysiology of infertility, assisted fertility and female reproduction. Infertility is a problem with a large magnitude. In this review we attempted to examine the various causes of female infertility and the role of OS in various etiologies of infertility. OS can arise as result of excessive production of free radicals and/or impaired antioxidant defense mechanism. An increasing number of published studies have pointed towards increased importance of the role of OS in female reproduction. Clearly, we have much to learn, but what we do know is that the role of OS in female reproduction cannot be underestimated. There is evidence that OS plays a role in conditions such as abortions, pre-eclampsia, hydatidiform mole, fetal embryopathies, preterm labor and pre-eclampsia and gestational diabetes, which lead to an immense burden of maternal and fetal morbidity and mortality. The review addresses the issue that both NOS and ROS species can lead to infertility problems and a spectrum of female reproductive disorders. We emphasize that free radicals have important physiological functions in the female reproductive tract as well as excessive free radicals precipitate female reproductive tract pathologies.

Reference values for ROS and NOS, minimum safe concentrations or physiologically beneficial concentrations have yet not been defined. Patients should be assessed according to the etiological factors and analyzed separately. Most of the published studies on oxidative stress are either observational or case control studies. Newer studies should be designed with more patient numbers; similar outcome parameters and uniform study populations so that results can be more easily compared. Measurement of OS in vivo is controversial. The sensitivity and specificity of various oxidative stress markers is not known. Measurement of biomarkers of OS is subject to interlaboratory variations, and interobserver differences. A uniform method with comprehensive assessment of the OS biomarkers should be used so that the results can be compared across the studies. Treatment strategies of antioxidant supplementation, directed toward reducing OS need to be investigated in randomized controlled trials. Antioxidants maybe advised when specific etiology cannot be identified as in idiopathic infertility as there is no other evidence based treatment for idiopathic infertility and reports indicate the presence of OS. Strategies to overcome OS in-vitro conditions and balancing between in vivo and in vitro environments can be utilized in ART, to successfully treat infertility. Interventions for overcoming oxidative stress in conditions such as abortions, preeclampsia, preterm labor and gestational diabetes and intrauterine growth retardation are still investigational with various randomized controlled trials in progress.

## Legend

Reprinted from an article in Reproductive BioMedicine Online by Agarwal and Allamaneni, 2004, with permission from Reproductive Healthcare Ltd [33].
